# Synthesis and crystal structures of three new benzotriazolylpropanamides

**DOI:** 10.1107/S2056989017007472

**Published:** 2017-05-26

**Authors:** Donna S. Amenta, Phil Liebing, Julia E. Biero, Robert J. Sherman, John W. Gilje, Frank T. Edelmann

**Affiliations:** a Chemistry Department, James Madison University, Harrisonburg, VA 22807, USA; b Chemisches Institut der Otto-von-Guericke-Universität Magdeburg, Universitätsplatz 2, 39106 Magdeburg, Germany

**Keywords:** crystal structure, acryl­amide, benzotriazole, benzotriazolylpropanamide, hydrogen bond, π–π stacking

## Abstract

The base-catalyzed Michael addition of 2-methyl­acryl­amide to benzotriazole afforded 3-(1*H*-benzotriazol-1-yl)-2-methyl­propanamide, C_10_H_12_N_4_O (**1**), in 32% yield in addition to small amounts of isomeric 3-(2*H*-benzotriazol-2-yl)-2-methyl­propanamide, C_10_H_12_N_4_O (**2**). In a similar manner, 3-(1*H*-benzotriazol-1-yl)-*N*,*N*-di­methyl­propanamide, C_11_H_14_N_4_O (**3**), was prepared from benzotriazole and *N*,*N*-di­methyl­acryl­amide. All three products have been structurally characterized by single-crystal X-ray diffraction. The crystal structures of **1** and **2** comprise infinite arrays formed by N—H⋯O and N—H⋯N bridges, as well as π–π inter­actions, while the mol­ecules of **3** are aggregated to simple π-dimers in the crystal.

## Abstract 2

The crystal structures of benzotriazolylpropanamides are governed by π–π stacking between the benzotriazolyl residues and, in the case of primary amide NH_2_ groups, by N—H⋯O and N—H⋯N bridging.

## Chemical context   

Di- and tridentate pyrazolyl-based ligands play an important role in the design of supra­molecular assemblies of metal complexes. Particularly notable among the large variety of such ligands are Trofimenko’s famous poly(pyrazol­yl)borates (‘scorpionates’) (Trofimenko, 1993 ▸, 2004 ▸; Marques *et al.*, 2002 ▸; Paulo *et al.*, 2004 ▸; Smith, 2008 ▸) and the poly(pyrazol­yl)methane ligands (Bassanetti *et al.*, 2016 ▸; Bigmore *et al.*, 2005 ▸; Krieck *et al.*, 2016 ▸; Otero *et al.*, 2013 ▸; Semeniuc & Reger, 2016 ▸). In a series of previous studies, we reported the synthesis and supra­molecular coordination chemistry of the simple, functionalized pyrazolyl-based ligand 3-(pyrazol-1-yl)prop­an­a­mide. This ligand is readily available in one step *via* base-catalyzed Michael addition of pyrazole to acryl­amide (Girma *et al.*, 2008 ▸). In combination with various first- and second-row transition metals (*e.g.* Mn, Fe, Ru, Co, Ni), 3-(1*H*-pyrazol-1-yl)propanamide allows the design of a variety of hydrogen-bonded supra­molecular assemblies, including different chains, sheets, and three-dimensional arrays (D’Amico *et al.*, 2015 ▸). As an additional advantage, the pyrazolylpropanamide ligand system can be easily modified either by attachment of substituents to the propanamide backbone (D’Amico *et al.*, 2015 ▸) or by replacing the pyrazole ring by other *N*-heterocycles such as triazole (D’Amico *et al.*, 2015 ▸; Wagner *et al.*, 2012 ▸). In our most recent study, we investigated the structural influence of benzotriazolyl as a hydro­phobic functional group, which imparts amphiphilic character to the ligand and forms the basis of novel supra­molecular assemblies. In the course of this work, the solid-state structures of 3-(1*H*-benzotriazol-1-yl)-propane­amide (= ‘BTPA’) and of several first-row transition metal complexes (Mn, Co, Cu) derived thereof have been described (Wang *et al.*, 2017 ▸). We report here the synthesis and structural characterization of three new potentially useful benzotriazolylpropanamide ligands.

**Figure 1:**
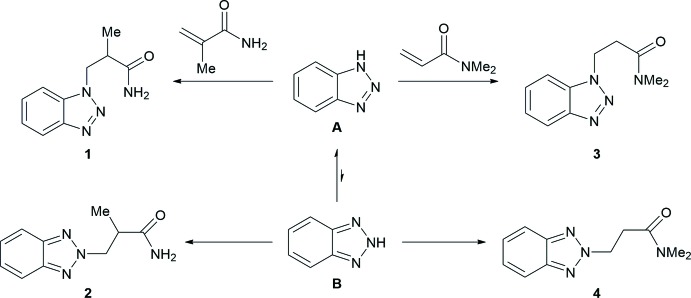
Formation of the 1*H*- and 2*H*-benzotriazolylpropanamides **1**–**4** from benzotriazole.

The title compounds were prepared by base-catalyzed Michael addition of benzotriazole to methyl-substituted acryl­amides, namely 2-methyl­acryl­amide and *N*,*N*-di­methyl­acryl­amide. As shown in the reaction scheme (Fig. 1 ▸), benzotriazole exists in two tautomeric forms **A** and **B**. Spectroscopic data (UV, IR and ^1^H NMR) (Negri & Caminati, 1996 ▸; Nesmeyanov *et al.*, 1969 ▸; Poznański *et al.*, 2007 ▸) and dipole moment measurements (Mauret *et al.*, 1974 ▸) revealed that the 1*H*-tautomer **A** is the predominant species at room temperature.

The thermal reaction of benzotriazole with 2-methyl­acryl­amide was carried out in the usual manner (D’Amico *et al.*, 2015 ▸; Wagner *et al.*, 2012 ▸; Wang *et al.*, 2017 ▸) in the presence of Triton B (= benzyl­tri­methyl­ammonium hydroxide) as basic catalyst. Repeated recrystallization of the crude product from ethanol afforded 3-(1*H*-benzotriazol-1-yl)-2-methyl­prop­an­amide (**1**) in 32% isolated yield. The compound was characterized through elemental analysis as well as IR and NMR (^1^H, ^13^C) spectroscopy. In the ^13^C NMR spectrum, the amide carbonyl C atom gives a characteristic resonance at 175.2 ppm. The formation of **1** as the main reaction product corresponds to the predominant presence of tautomer **A** in the starting benzotriazole. From the mother liquor of the recrystallization of **1**, a small amount of colorless crystals could be isolated, which were found to be the isomer 3-(2*H*-benzotriazol-2-yl)-2-methyl­propanamide (**2**) resulting from the reaction of the 2*H*-tautomer **B** with 2-methyl­acryl­amide. Compound **2** could also be fully characterized by elemental analysis as well as IR and NMR data.
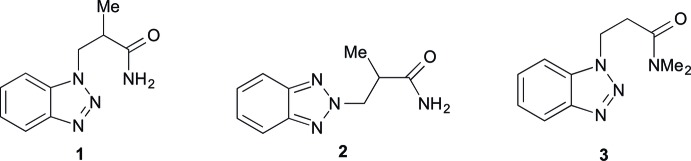


In a similar manner, a reaction of benzotriazole with neat *N*,*N*-di­methyl­acryl­amide in the presence of Triton B afforded a yellow oil which was shown to be an approximate 2:1 mixture of **3** and **4**. Once again, the main component was the Michael addition product resulting from the 1*H*-tautomer **A** of benzotriazole. Thus far, only isomer **3** could be isolated in pure form by recrystallization of the oily crude product from ethanol. The identity of 3-(1*H*-benzotriazol-1-yl)-*N*,*N*-di­methyl­propanamide **3** was confirmed by elemental analysis and spectroscopic data (IR, ^1^H and ^13^C NMR). In the ^13^C NMR spectrum, the NMe_2_ group gives rise to two resonances at δ 33.2 and 35.5 ppm, whereas the signal of the amide carbonyl C atom is found at δ 169.5 ppm.

## Structural commentary   

**Figure 2:**
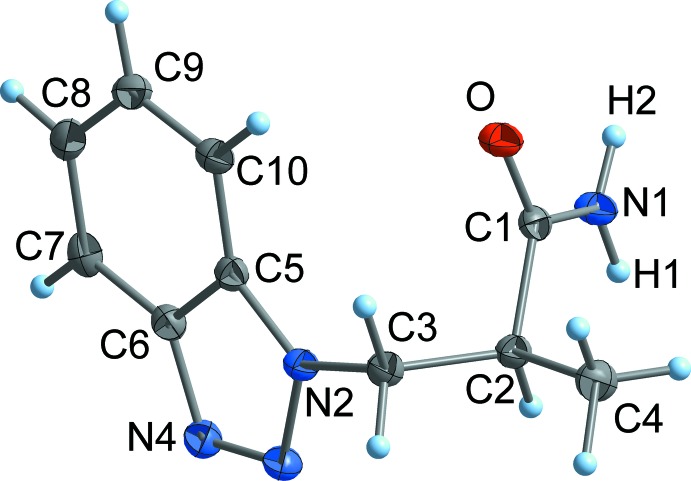
The mol­ecular structure of **1** in the crystal. Displacement ellipsoids are drawn at the 50% probability level.

Compounds **1**–**3** exist as well-defined monomeric mol­ecules in the crystal, without any solvent of crystallization (Figs. 2 ▸–4 ▸ ▸). The C=O separations are in a narrow range around 1.24 Å and are therefore virtually equal with those observed in related functionalized propanamides (Girma *et al.* 2008 ▸; Wagner *et al.* 2012 ▸; D’Amico *et al.* 2015 ▸; Wang *et al.* 2017 ▸). Thus, the C=O distance is not markedly influenced by hydrogen bonding, as there are N—H⋯O bridges in **1** and **2**, but not in **3** (see *Supra­molecular features* section). The same applies to the amide C—N separation, which is around 1.33 Å in all compounds. The torsion angle C1—C2—C3—N between the amide group and the 1*H*-benzotriazol-1-yl residue is 71.0 (1)° (**1**) and −72.2 (2)° (**3**), respectively, which is close to the value observed in the unsubstituted BTPA (71.3 (1)°; Wang *et al.*, 2017 ▸). By contrast, the same torsion angle in the 2*H*-benzotriazole-derived compound **2** is considerably smaller at 59.7 (1)°.

## Supra­molecular features   

In **1** and **2**, the mol­ecules are inter­connected to dimeric subunits by 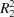
(8)-type N—H⋯O bridges, which is a very typical motif (Bernstein *et al.*, 1995 ▸). These amide dimers are again inter­connected by N—H⋯N bridges (Tables 1 ▸ and 2 ▸) between the remaining amide N—H moiety and the benzotriazolyl group, resulting in an infinite chain of rings in both cases. In **1**, the dimeric subunits are linked by a 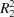
(16) bridge to N4 (Fig. 5 ▸), while a *C*(7) bridge involving N2 is realized in compound **2** (Fig. 7 ▸). The latter leads to an 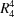
(18) motif at the binary level. The hydrogen-bridge pattern in **1** and **2** is therefore entirely different than in the unbridged BTPA, where supra­molecular layers are formed exclusively by N—H⋯O bridges (Wang *et al.*, 2017 ▸). As has been discussed for BTPA and its metal complexes, the N—H⋯N bonds are significantly weaker than the N—H⋯O bonds. Both the N⋯O separation [**1**: N1⋯O 2.897 (1) Å; **2**: N1⋯O 2.875 (2) Å] and the N⋯N separations [**1**: N1⋯N4 3.002 (1) Å; **2**: N1⋯N2 3.085 (2) Å] are in the typical range. In the crystal structure of **3**, no hydrogen bonds are present as the amide H atoms are replaced by methyl groups.

In both **1** and **2**, the supra­molecular chains are further aggregated by π–π inter­actions between the benzotriazolyl rings. In **1**, a three-dimensional framework is present (Fig. 6 ▸), where two different types of π inter­actions can be distinguished. First, the C_6_ rings of each two adjoining benzotriazolyl groups are stacked in a typical parallel-displaced fashion (*cf.* Fig. 10 ▸ *a*). The shortest C⋯C contact is 3.364 (2) Å between C7 and C9 and the distance between the C_6_ ring centroids is 3.655 (2) Å, which is in the range of strong π inter­actions (McGaughey *et al.*, 1998 ▸). The so-formed π dimers are inter­connected by another π inter­action to an infinite chain, where an attractive inter­action seems to exist between the whole bicyclic C_6_N_3_ system rather than between the C_6_ rings only (*cf.* Fig. 10 ▸ *c*). The closest inter­molecular separations are 3.308 (2) Å (C9⋯N2) and 3.403 (2) Å (C5⋯C10), and therefore in the same range as in the former mentioned inter­action. In the case of **2**, a layer structure parallel to (001) is formed (Fig. 8 ▸). The geometry of the inter­action between the C_6_ rings is similar as in **1**, but the closest C⋯C contact exists between C5 and C9 with 3.521 (2) Å, and the corresponding separation between the C_6_ centroids is considerably larger at 3.933 (2) Å (*cf.* Fig. 10 ▸ *b*). In **3**, only two mol­ecules are stacked together to a simple π dimer (Fig. 9 ▸), with participation of the whole C_6_N_3_ bicycle similar as described above for **1** (*cf.* Fig. 10 ▸ *c*). Here, the closest inter­molecular contacts are 3.468 (2) Å (C8⋯N2) and 3.509 (2) Å (C4⋯C9), which is significantly larger than in **1**. Comparable π inter­actions as in **1**–**3** have not been observed in the unbridged BTPA, but in its metal complexes [*M*Cl_2_(BTPA)_2_] (*M* = Mn, Co, Cu; min. C⋯C 3.45 Å; Wang *et al.*, 2017 ▸). The arrangement of the benzotriazolyl groups in the latter compounds is similar to that in **3** (*cf.* Fig. 10 ▸ *c*).

## Database survey   

For reviews on di- and tridentate pyrazolyl-based ligands, see Bassanetti *et al.* (2016 ▸), Bigmore *et al.* (2005 ▸), Krieck *et al.* (2016 ▸), Marques *et al.* (2002 ▸), Otero *et al.* (2013 ▸), Paulo *et al.* (2004 ▸), Semeniuc & Reger (2016 ▸), Smith (2008 ▸), Trofimenko (1993 ▸, 2004 ▸).

For the tautomerism of benzotriazole, see Mauret *et al.* (1974 ▸), Negri & Caminati (1996 ▸); Nesmeyanov *et al.* (1969 ▸), Poznański *et al.* (2007 ▸).

For other structurally characterized 3-pyrazolylpropanamide-derived ligands, see D’Amico *et al.* (2015 ▸), Girma *et al.* (2008 ▸), Wagner *et al.* (2012 ▸), Wang *et al.* (2017 ▸).

## Synthesis and crystallization   

All manipulations were performed under inert nitro­gen or argon atmospheres using standard Schlenk techniques or in a Vacuum Atmospheres Glove Box. The starting materials were obtained from commercial sources and used as received. Solvents were dried using an Innovative Technology, Inc, solvent purification system. Microanalysis was performed by Galbraith Laboratories, Inc, Knoxville, TN, USA. NMR spectra were obtained using Bruker Avance 300 MHz and 400 MHz NMR Spectrometers. IR spectra were recorded using KBr pellets with a ThermoNicolet Avatar 370 FT–IR between 4000 cm^−1^ and 400 cm^−1^.

*Preparation of 2-methyl-3-(1H-benzotriazol-1-yl)propan­amide (**1**) and 2-methyl-3-(2H-benzotriazol-2-yl)propanamide (**2**):*

In a 150 mL three-neck flask, a mixture of benzotriazole (5.032 g, 42.24 mmol), 2-methyl acryl­amide (3.731 g, 43.84 mmol) and 2 mL of Triton B was heated for 6.5 h in a boiling water bath. The mixture solidified upon cooling. The crude product was slurried with 95% ethanol and the remaining solid recrystallized three times from 95% ethanol to yield 2.841g (13.91 mmol, 32%) of spectroscopically pure **1**. Single crystals suitable for X-ray diffraction were obtained from these recrystallizations. M.p. 476–479 K. Analysis calculated for C_10_H_12_N_4_O, *M* = 204.20 g mol^−1^: C 58.82; H 5.92; N 27.44. Found: C 58.73; H 5.96; N 27.72. IR (KBr, cm^−1^): 3307 *vs*, 3208 *s*, 3155 *vs*, 2968 *m*, 2930 *w*, 1685 *vs*, 1442 *m*, 1315 *m*, 1226 *s*, 780 *m*, 742 *vs*. ^1^H NMR (400 MHz, DMSO-*d*_6_): 1.07 (*d*, *J*_2-4_ = 7 Hz, 3H; C*H*_3_), 3.06 (*sext*, *J*_2-4_ = 7 Hz, *J*_2-3_ = 7 Hz, 1H; 2-C*H*), 4.61 (*dd*, *J*_2-3_ = 7 Hz, *J*_2-2′_ = 14 Hz, 1H; C*H*_2_), 4.86 (*dd*, *J*_2-3_ = 7 Hz, *J*_2-2′_ = 14 Hz, 1H; C*H*_2_), 6.88 (*s br*, 1H; N*H*), 7.39 (*m*, 1H; 8-C*H* or 9-C*H*), 7.42 (*s br*, 1H; N*H*) 7.54 (*m*, 1H; 8-C*H* or 9-C*H*), 7.87 (*m*, 1H; 7-C*H* or 10-C*H*), 8.02 (*m*, 1H; 7-C*H* or 10-C*H*) ppm. The resonances for positions 7–10 appear as multiplets that can be inter­preted if the coupling constants between adjacent protons are 7–8 Hz, with longer range couplings of about 1 Hz. ^13^C{^1^H} NMR (100 MHz, DMSO-*d*_6_): 16.2 (*C*H_3_), 40.6 (2-*C*H), 50.6 (*C*H_2_), 111.5 (10-*C*H), 119.4 (7-*C*H), 124.3 (8-*C*H), 127.5 (9-*C*H), 133.5 (5-*C*), 145.4 (6-*C*), 175.3 (*C*O) (for numbering scheme *cf.* Fig. 2 ▸).

**Figure 3:**
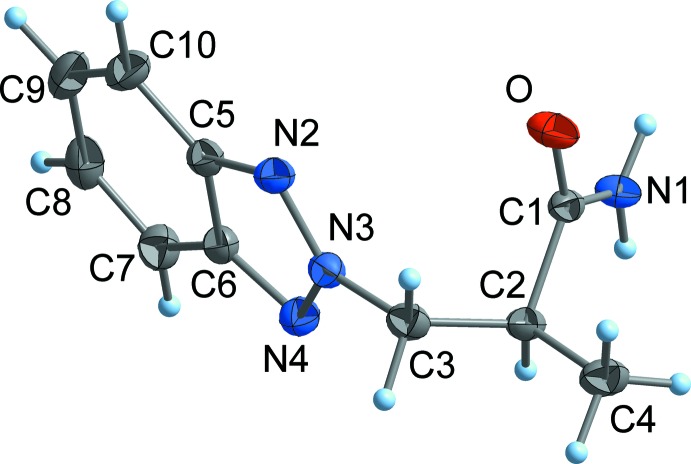
The mol­ecular structure of **2** in the crystal. Displacement ellipsoids are drawn at the 50% probability level.

The mother liquor remaining after the isolation of **1** was concentrated, and two additional crops of crystals were obtained. The second crop was several milligrams of nearly pure **2** and contained crystals suitable for X-ray diffraction. M.p. 476–479 K. Analysis calculated for C_10_H_12_N_4_O, *M* = 204.20 g mol^−1^: C 58.82; H 5.92; N 27.44. Found: C 58.92; H 6.20; N 27.50. IR (KBr, cm^−1^): 3307 *vs*, 3208 *s*, 3155 *vs*, 2968 *m*, 2930 *w*, 1685 *vs*, 1442 *m*, 1315 *m*, 1226 *s*, 780 *m*, 742 *vs*. ^1^H NMR (400 MHz, DMSO-*d*_6_): 1.06 (*d*, *J*_2-4_ = 7.0 Hz, 3H; C*H*_3_), 3.06 (*sext*, *J*_2-4_ = 7.0 Hz, *J*_2-3_ = 7.0 Hz, *J*_2-3′_ = 7.7 Hz, 1H; 2-C*H*), 4.64 (*dd*, *J*_2-3_ = 7.0 Hz, *J*_3-3′_ = 13.3 Hz, 1H; C*H*_2_), 4.93 (*dd*, *J*_2-3_ = 7.7 Hz, *J*_3-3′_ = 13.3 Hz, 1H; C*H*_2_), 6.91 (*s br*, 1H; N*H*), 7.43 (*m*, 2H; 6,9-C*H*), 7.48 (*s br*; N*H*), 7.91 (*m*, 2H; 7,8-C*H*). The resonances for 6-C*H*, 7-C*H*, 8-C*H* and 9-C*H* appear as an AA’BB’ pattern. While there is no unique solution for *AA*′*BB*′ spectra, the ^1^H spectrum of the aromatic region of **2** can be duplicated using reasonable values of the coupling constants: *J*_7-8_ = 6.8 Hz, *J*_6-7_ = *J*_8-9_ = 8.6 Hz, *J*_6-8_ = *J*_7-9_ = 1.0 Hz, and *J*_6-9_ = 1.0 Hz. ^13^C{^1^H} NMR (100 MHz, DMSO-*d*_6_): 16.2 (*C*H_3_), 40.5 (2-*C*H), 58.5 (*C*H_2_), 118.3 (6,9-*C*H), 126.8 (7,8-*C*H), 144.1 (5,10-*C*), 175.0 (*C*O) (for numbering scheme *cf.* Fig. 3 ▸).

*Preparation of N,N-dimethyl-3-(1H-benzotriazol-1-yl)propanamide (**3**):*

**Figure 4:**
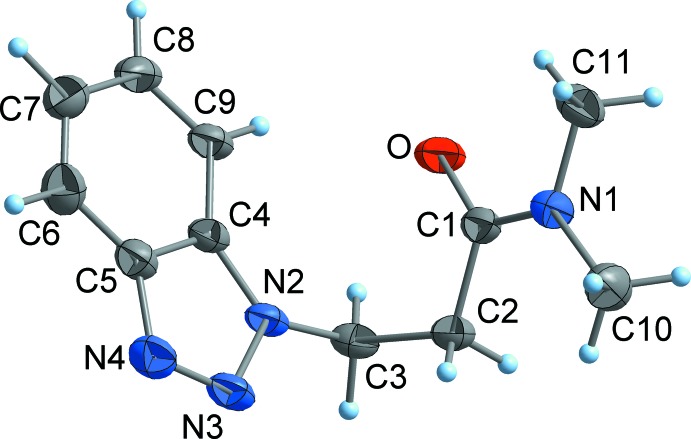
The mol­ecular structure of **3** in the crystal. Displacement ellipsoids are drawn at the 50% probability level. The methyl group C11 shows rotational disorder over two orientations (only one orientation of the H atoms is shown).

**Figure 5:**
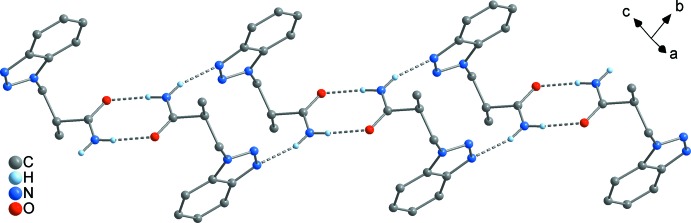
Supra­molecular chain of rings in **1**, formed by N—H⋯O and N—H⋯N bridging.

**Figure 6:**
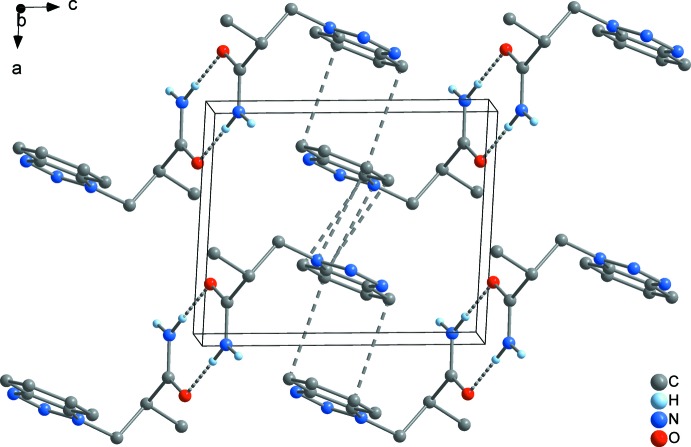
The unit cell of **1**, illustrating the aggregation of the chains shown in Fig. 5 ▸ by π–π stacking into a three-dimensional framework, viewed in a projection on (010).

**Figure 7:**
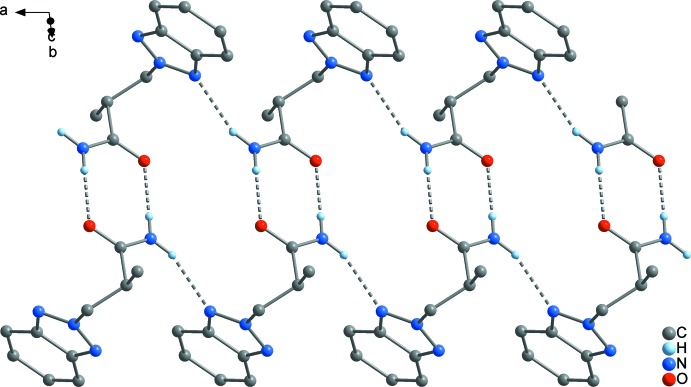
Supra­molecular chain of rings in **2**, formed by N—H⋯O and N—H⋯N bridging, extending along the crystallographic *a* axis.

**Figure 8:**
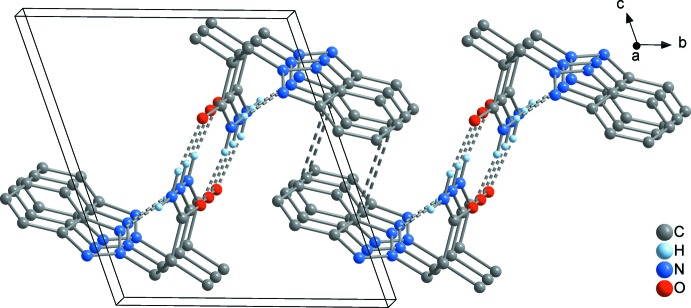
The unit cell of **2**, illustrating the aggregation of the chains shown in Fig. 6 ▸ by π–π stacking, to a two-dimensional array extending parallel to (001), viewed in a projection on (100).

**Figure 9:**
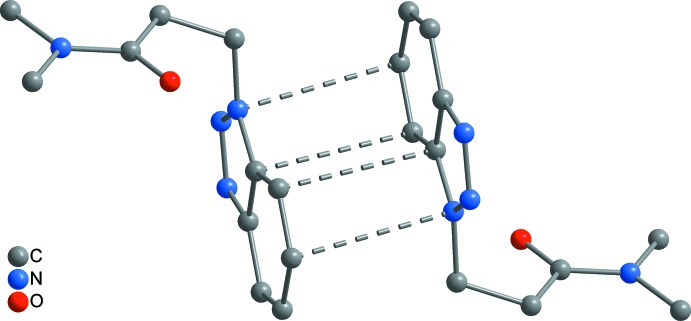
Supra­molecular dimer of **3**, formed by π–π stacking.

**Figure 10:**
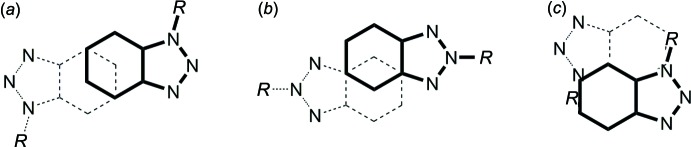
Comparison of the arrangement of the benzotriazolyl rings in the crystal structures of 3-benzotriazolyl­propan­amides: stacking of C_6_ rings in **1** (*a*) and in **2** (*b*), stacking of C_6_N_3_ bicycles in **1**, **3** and in [*M*Cl_2_(BTPA)_2_] (*M* = Mn, Co, Cu) (*c*), each viewed in a projection on the C_6_N_3_ plane.

In a 150 mL three-neck flask, a mixture of benzotriazole (5.99 g, 50.0 mmol), *N*,*N*-di­methyl­acryl­amide (4.78 g, 48.8 mmol) and 2 mL of Triton B was heated for 6.5 h in a boiling water bath under nitro­gen. Upon cooling to 278 K, a yellow oil was obtained. This mixture was an approximate 2:1 mixture of **3** and **4**. After recrystallization from ethanol, samples of pure **3** could be obtained. Single crystals suitable for X-ray diffraction were obtained from recrystallization from a CHCl_3_/hexa­nes mixture_._ M.p. 338–339 K. Analysis calculated for C_10_H_12_N_4_O, *M* = 218.26 g mol^−1^: C 60.53; H 6.47; N 25.60. Found: C 60.48; H 6.27; N 25.87. IR (KBr, cm^−1^): 3082 *w*, 3050 *w*, 3015 *w*, 2967 *m*, 2939 *m*, 2911 *m*, 1644 *vs*, 1496 *s*, 1452 *s*, 1414 *s*, 1396 *s*, 1338 *m*, 1298 *m*, 1216 *s*, 1151 *s*, 1092 *s*, 942 *m*, 761 *s*, 743 *vs*. ^1^H NMR (300 MHz, CDCl_3_): 2.92 (*s*, 3H; NC*H*_3_), 2.94 (*s*; NC*H*_3_), 3.14 (*t*, *J*_2-3_ = 7 Hz, 2H; 2-C*H*_2_), 4.98 (*t*, *J*_2-3_ = 7 Hz, 2H; 3-C*H*_2_), 7.38 (*m*, 2H; 7-C*H* or 8-C*H*), 7.51 (*m*, 2H; 7-C*H* or 8-C*H*), 7.20 (*m*, 2H; 6-C*H* or 9-C*H*), 8.05 (*m*, 2H; 6-C*H* or 9-C*H*). The resonances for positions 6–9 appear as multiplets that can be inter­preted if the coupling constants between adjacent protons are 7–8 Hz, with longer range couplings of about 1 Hz. ^13^C{^1^H} NMR (100 MHz, CDCl_3_): 33.2 (N*C*H_3_), 35.5 (N*C*H_3_), 37.0 (2-*C*H_2_), 43.9 (3-*C*H_2_), 110.0 (9-*C*H), 119.7 (6-*C*H), 123.9 (7-*C*H), 127.4 (8-*C*H), 133.3 (4-*C*), 145.8 (5-*C*), 169.5 (*C*O) (for numbering scheme *cf.* Fig. 4 ▸).

## Refinement   

Crystal data, data collection and structure refinement details are summarized in Table 3 ▸. All H atoms were fixed geom­etrically using a riding model with *U*_iso_(H) = 1.2 *U*_eq_(*X*) (*X* = C, N). The CH_3_ groups were allowed to rotate freely around the C—*X* vector (*X* = C, N) (AFIX 137 in SHELXL), and the amide NH_2_ groups in **1** and **2** were constrained to be planar (AFIX 93 in SHELXL). C—H distances in CH_3_ groups were constrained to 0.98 Å, those in CH_2_ groups to 0.99 Å and those in CH groups to 1.00 Å. N—H distances in **1** and **2** were constrained to 0.88 Å. For compound **2**, reflection (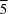
62) strongly disagreed with the structural model and was therefore omitted from the refinement. In the case of compound **3**, one N-bonded methyl group (C11) was refined as rotationally disordered over two positions. Site occupancy factors were refined freely to 0.59 (2) for H12*A*, H13*A* and H14*A*, and to 0.41 (2) for H12*B*, H13*B* and H14*B*.

## Supplementary Material

Crystal structure: contains datablock(s) 1, 2, 3. DOI: 10.1107/S2056989017007472/zl2702sup1.cif

Structure factors: contains datablock(s) 1. DOI: 10.1107/S2056989017007472/zl27021sup2.hkl

Supporting information file. DOI: 10.1107/S2056989017007472/zl27021sup5.cml

Structure factors: contains datablock(s) 2. DOI: 10.1107/S2056989017007472/zl27022sup3.hkl

Supporting information file. DOI: 10.1107/S2056989017007472/zl27022sup6.cml

Structure factors: contains datablock(s) 3. DOI: 10.1107/S2056989017007472/zl27023sup4.hkl

Supporting information file. DOI: 10.1107/S2056989017007472/zl27023sup7.cml

CCDC references: 1550158, 1550157, 1550156

Additional supporting information:  crystallographic information; 3D view; checkCIF report

## Figures and Tables

**Table 1:** Hydrogen-bond geometry (Å, °) for **1** [Chem scheme1]

*D*—H⋯*A*	*D*—H	H⋯*A*	*D*⋯*A*	*D*—H⋯*A*
N1—H2⋯O^i^	0.88	2.02	2.8970 (12)	175
N1—H1⋯N4^ii^	0.88	2.16	3.0017 (14)	161

Symmetry codes: (i) 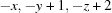; (ii) 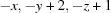.

**Table 2:** Hydrogen-bond geometry (Å, °) for **2** [Chem scheme1]

*D*—H⋯*A*	*D*—H	H⋯*A*	*D*⋯*A*	*D*—H⋯*A*
N1—H1⋯O^i^	0.88	2.00	2.8745 (18)	170
N1—H2⋯N2^ii^	0.88	2.24	3.0850 (18)	161

Symmetry codes: (i) 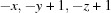; (ii) 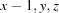.

**Table 3:** Experimental details

	**1**	**2**	**3**
Crystal data			
Chemical formula	C_10_H_12_N_4_O	C_10_H_12_N_4_O	C_11_H_14_N_4_O
*M*_r_	204.24	204.24	218.26
Crystal system, space group	Triclinic, *P* 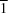	Triclinic, *P* 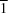	Triclinic, *P* 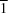
Temperature (K)	100	133	153
*a*, *b*, *c* (Å)	7.3885 (9), 8.072 (1), 9.2976 (13)	5.5961 (11), 9.3462 (19), 10.472 (2)	7.1732 (6), 7.9945 (6), 9.5912 (7)
α, β, γ (°)	69.039 (12), 89.498 (10), 75.915 (10)	109.83 (3), 90.93 (3), 97.14 (3)	83.910 (6), 86.247 (6), 81.528 (6)
*V* (Å^3^)	500.37 (12)	510.2 (2)	540.25 (7)
*Z*	2	2	2
Radiation type	Cu *K*α	Mo *K*α	Mo *K*α
μ (mm^−1^)	0.76	0.09	0.09
Crystal size (mm)	0.15 × 0.10 × 0.08	0.48 × 0.33 × 0.25	0.34 × 0.32 × 0.28

Data collection			
Diffractometer	Agilent Xcalibur, Atlas, Nova	Stoe IPDS 2T	Stoe IPDS 2T
Absorption correction	Multi-scan (*CrysAlis PRO*, Agilent, 2003 ▸)	–	–
*T*_min_, *T*_max_	0.919, 1.000	–	–
No. of measured, independent and observed [*I* > 2σ(*I*)] reflections	28193, 2053, 2012	3731, 1774, 1653	4194, 1904, 1596
*R*_int_	0.026	0.056	0.062
(sin θ/λ)_max_ (Å^−1^)	0.626	0.595	0.595

Refinement			
*R*[*F* ^2^ > 2σ(*F* ^2^)], *wR*(*F* ^2^), *S*	0.032, 0.081, 1.11	0.037, 0.087, 1.09	0.047, 0.131, 1.03
No. of reflections	2053	1774	1904
No. of parameters	138	138	149
H-atom treatment	H-atom parameters constrained	H-atom parameters constrained	H-atom parameters constrained
Δρ_max_, Δρ_min_ (e Å^−3^)	0.33, −0.21	0.25, −0.18	0.22, −0.20

Computer programs: *CrysAlis PRO* (Agilent, 2003 ▸), *X-AREA* and *X-RED* (Stoe & Cie, 2002 ▸), *SHELXS97* (Sheldrick, 2008 ▸), *SIR97* (Altomare *et al.*, 1999 ▸), *SHELXL2016* (Sheldrick, 2015 ▸) and *DIAMOND* (Brandenburg, 1999 ▸).

## supplementary crystallographic information

### (1) 3-(1H-Benzotriazol-1-yl)-2-methylpropanamide Crystal data

**Table:**

C_10_H_12_N_4_O	*Z* = 2
*M**_r_* = 204.24	*F*(000) = 216
Triclinic, *P*1	*D*_x_ = 1.356 Mg m^−^^3^
*a* = 7.3885 (9) Å	Cu *K*α radiation, λ = 1.54184 Å
*b* = 8.072 (1) Å	Cell parameters from 21215 reflections
*c* = 9.2976 (13) Å	θ = 5.1–76.1°
α = 69.039 (12)°	µ = 0.76 mm^−^^1^
β = 89.498 (10)°	*T* = 100 K
γ = 75.915 (10)°	Prism, colorless
*V* = 500.37 (12) Å^3^	0.15 × 0.10 × 0.08 mm

### (1) 3-(1H-Benzotriazol-1-yl)-2-methylpropanamide Data collection

**Table:**

Agilent Xcalibur, Atlas, Nova diffractometer	2053 independent reflections
Radiation source: fine-focus sealed tube	2012 reflections with *I* > 2σ(*I*)
Detector resolution: 10.3543 pixels mm^-1^	*R*_int_ = 0.026
ω scans	θ_max_ = 75.0°, θ_min_ = 5.1°
Absorption correction: multi-scan (CrysAlis PRO, Agilent, 2003)	*h* = −9→9
*T*_min_ = 0.919, *T*_max_ = 1.000	*k* = −10→10
28193 measured reflections	*l* = −9→11

### (1) 3-(1H-Benzotriazol-1-yl)-2-methylpropanamide Refinement

**Table:**

Refinement on *F*^2^	Secondary atom site location: difference Fourier map
Least-squares matrix: full	Hydrogen site location: inferred from neighbouring sites
*R*[*F*^2^ > 2σ(*F*^2^)] = 0.032	H-atom parameters constrained
*wR*(*F*^2^) = 0.081	*w* = 1/[σ^2^(*F*_o_^2^) + (0.0349*P*)^2^ + 0.1806*P*] where *P* = (*F*_o_^2^ + 2*F*_c_^2^)/3
*S* = 1.11	(Δ/σ)_max_ < 0.001
2053 reflections	Δρ_max_ = 0.33 e Å^−^^3^
138 parameters	Δρ_min_ = −0.21 e Å^−^^3^
0 restraints	Extinction correction: SHELXL2016 (Sheldrick, 2015), Fc^*^=kFc[1+0.001xFc^2^λ^3^/sin(2θ)]^-1/4^
Primary atom site location: structure-invariant direct methods	Extinction coefficient: 0.0117 (12)

### (1) 3-(1H-Benzotriazol-1-yl)-2-methylpropanamide Special details

**Table:**

Geometry. All esds (except the esd in the dihedral angle between two l.s. planes) are estimated using the full covariance matrix. The cell esds are taken into account individually in the estimation of esds in distances, angles and torsion angles; correlations between esds in cell parameters are only used when they are defined by crystal symmetry. An approximate (isotropic) treatment of cell esds is used for estimating esds involving l.s. planes.

### (1) 3-(1H-Benzotriazol-1-yl)-2-methylpropanamide Fractional atomic coordinates and isotropic or equivalent isotropic displacement parameters (Å^2^)

**Table:**

	*x*	*y*	*z*	*U*_iso_*/*U*_eq_	
C1	0.15133 (14)	0.68751 (14)	0.89673 (11)	0.0176 (2)	
C2	0.25594 (14)	0.83380 (13)	0.82012 (11)	0.0179 (2)	
H3	0.165006	0.949315	0.752342	0.022*	
C3	0.40506 (14)	0.76911 (14)	0.72364 (11)	0.0184 (2)	
H4	0.488091	0.853320	0.694399	0.022*	
H5	0.482564	0.645326	0.787098	0.022*	
C4	0.35221 (17)	0.87131 (16)	0.94623 (13)	0.0262 (3)	
H8	0.258168	0.911025	1.010223	0.031*	
H6	0.416372	0.967988	0.898144	0.031*	
H7	0.443677	0.758907	1.011003	0.031*	
C5	0.29153 (13)	0.61763 (14)	0.55521 (12)	0.0167 (2)	
C6	0.21574 (14)	0.69407 (14)	0.40143 (12)	0.0181 (2)	
C7	0.16602 (14)	0.58415 (15)	0.32859 (12)	0.0213 (2)	
H9	0.113426	0.634772	0.224400	0.026*	
C8	0.19688 (14)	0.40025 (15)	0.41480 (13)	0.0220 (2)	
H10	0.165146	0.321802	0.369143	0.026*	
C9	0.27490 (14)	0.32485 (14)	0.57016 (13)	0.0214 (2)	
H11	0.294873	0.196704	0.625394	0.026*	
C10	0.32283 (14)	0.43018 (14)	0.64405 (12)	0.0191 (2)	
H12	0.373910	0.379176	0.748680	0.023*	
N1	−0.03436 (12)	0.74707 (12)	0.89024 (10)	0.0209 (2)	
H2	−0.101453	0.669647	0.936062	0.025*	
H1	−0.089861	0.863817	0.840137	0.025*	
N2	0.32450 (12)	0.76231 (11)	0.58395 (10)	0.0173 (2)	
N3	0.27302 (13)	0.91737 (12)	0.45699 (10)	0.0209 (2)	
N4	0.20701 (13)	0.87851 (12)	0.34604 (10)	0.0213 (2)	
O	0.23854 (10)	0.52560 (10)	0.96498 (9)	0.02255 (19)	

### (1) 3-(1H-Benzotriazol-1-yl)-2-methylpropanamide Atomic displacement parameters (Å^2^)

**Table:**

	*U*^11^	*U*^22^	*U*^33^	*U*^12^	*U*^13^	*U*^23^
C1	0.0225 (5)	0.0179 (5)	0.0130 (4)	−0.0063 (4)	0.0019 (4)	−0.0056 (4)
C2	0.0214 (5)	0.0164 (5)	0.0158 (5)	−0.0061 (4)	0.0012 (4)	−0.0049 (4)
C3	0.0195 (5)	0.0197 (5)	0.0171 (5)	−0.0071 (4)	0.0007 (4)	−0.0065 (4)
C4	0.0344 (6)	0.0286 (6)	0.0207 (5)	−0.0144 (5)	0.0023 (4)	−0.0110 (4)
C5	0.0152 (4)	0.0180 (5)	0.0173 (5)	−0.0046 (4)	0.0030 (4)	−0.0068 (4)
C6	0.0171 (5)	0.0187 (5)	0.0163 (5)	−0.0030 (4)	0.0026 (4)	−0.0048 (4)
C7	0.0190 (5)	0.0278 (6)	0.0182 (5)	−0.0050 (4)	0.0021 (4)	−0.0103 (4)
C8	0.0200 (5)	0.0259 (5)	0.0258 (6)	−0.0083 (4)	0.0055 (4)	−0.0148 (4)
C9	0.0209 (5)	0.0178 (5)	0.0256 (5)	−0.0063 (4)	0.0053 (4)	−0.0071 (4)
C10	0.0187 (5)	0.0182 (5)	0.0178 (5)	−0.0049 (4)	0.0021 (4)	−0.0035 (4)
N1	0.0207 (4)	0.0165 (4)	0.0213 (4)	−0.0050 (3)	0.0022 (3)	−0.0018 (3)
N2	0.0206 (4)	0.0152 (4)	0.0148 (4)	−0.0053 (3)	0.0018 (3)	−0.0037 (3)
N3	0.0255 (5)	0.0170 (4)	0.0170 (4)	−0.0046 (3)	0.0022 (3)	−0.0032 (3)
N4	0.0247 (5)	0.0195 (4)	0.0170 (4)	−0.0037 (4)	0.0007 (3)	−0.0050 (3)
O	0.0222 (4)	0.0172 (4)	0.0234 (4)	−0.0045 (3)	0.0025 (3)	−0.0020 (3)

### (1) 3-(1H-Benzotriazol-1-yl)-2-methylpropanamide Geometric parameters (Å, º)

**Table:**

C1—O	1.2369 (13)	C5—C10	1.4014 (14)
C1—N1	1.3329 (14)	C6—N4	1.3752 (13)
C1—C2	1.5271 (14)	C6—C7	1.4049 (15)
C2—C3	1.5261 (14)	C7—C8	1.3730 (15)
C2—C4	1.5316 (14)	C7—H9	0.9500
C2—H3	1.0000	C8—C9	1.4157 (16)
C3—N2	1.4576 (13)	C8—H10	0.9500
C3—H4	0.9900	C9—C10	1.3744 (15)
C3—H5	0.9900	C9—H11	0.9500
C4—H8	0.9800	C10—H12	0.9500
C4—H6	0.9800	N1—H2	0.8800
C4—H7	0.9800	N1—H1	0.8800
C5—N2	1.3628 (13)	N2—N3	1.3505 (12)
C5—C6	1.3977 (14)	N3—N4	1.3096 (13)
			
O—C1—N1	123.48 (9)	N4—C6—C5	108.40 (9)
O—C1—C2	120.43 (9)	N4—C6—C7	130.82 (10)
N1—C1—C2	116.03 (9)	C5—C6—C7	120.77 (10)
C3—C2—C1	110.96 (8)	C8—C7—C6	116.99 (10)
C3—C2—C4	108.61 (9)	C8—C7—H9	121.5
C1—C2—C4	108.83 (8)	C6—C7—H9	121.5
C3—C2—H3	109.5	C7—C8—C9	121.51 (10)
C1—C2—H3	109.5	C7—C8—H10	119.2
C4—C2—H3	109.5	C9—C8—H10	119.2
N2—C3—C2	112.50 (8)	C10—C9—C8	122.44 (10)
N2—C3—H4	109.1	C10—C9—H11	118.8
C2—C3—H4	109.1	C8—C9—H11	118.8
N2—C3—H5	109.1	C9—C10—C5	115.74 (10)
C2—C3—H5	109.1	C9—C10—H12	122.1
H4—C3—H5	107.8	C5—C10—H12	122.1
C2—C4—H8	109.5	C1—N1—H2	120.0
C2—C4—H6	109.5	C1—N1—H1	120.0
H8—C4—H6	109.5	H2—N1—H1	120.0
C2—C4—H7	109.5	N3—N2—C5	110.44 (8)
H8—C4—H7	109.5	N3—N2—C3	119.41 (8)
H6—C4—H7	109.5	C5—N2—C3	130.14 (8)
N2—C5—C6	104.07 (9)	N4—N3—N2	108.81 (8)
N2—C5—C10	133.36 (9)	N3—N4—C6	108.28 (8)
C6—C5—C10	122.55 (10)		
			
O—C1—C2—C3	47.10 (12)	C8—C9—C10—C5	−0.69 (15)
N1—C1—C2—C3	−135.56 (9)	N2—C5—C10—C9	−177.82 (10)
O—C1—C2—C4	−72.35 (12)	C6—C5—C10—C9	0.27 (15)
N1—C1—C2—C4	104.99 (10)	C6—C5—N2—N3	−0.07 (11)
C1—C2—C3—N2	70.99 (10)	C10—C5—N2—N3	178.28 (11)
C4—C2—C3—N2	−169.42 (8)	C6—C5—N2—C3	−179.08 (9)
N2—C5—C6—N4	0.01 (11)	C10—C5—N2—C3	−0.74 (18)
C10—C5—C6—N4	−178.56 (9)	C2—C3—N2—N3	82.84 (11)
N2—C5—C6—C7	178.91 (9)	C2—C3—N2—C5	−98.22 (12)
C10—C5—C6—C7	0.34 (15)	C5—N2—N3—N4	0.11 (11)
N4—C6—C7—C8	178.10 (10)	C3—N2—N3—N4	179.24 (8)
C5—C6—C7—C8	−0.53 (15)	N2—N3—N4—C6	−0.10 (11)
C6—C7—C8—C9	0.13 (15)	C5—C6—N4—N3	0.06 (11)
C7—C8—C9—C10	0.51 (16)	C7—C6—N4—N3	−178.70 (10)

### (1) 3-(1H-Benzotriazol-1-yl)-2-methylpropanamide Hydrogen-bond geometry (Å, º)

**Table:**

*D*—H···*A*	*D*—H	H···*A*	*D*···*A*	*D*—H···*A*
N1—H2···O^i^	0.88	2.02	2.8970 (12)	175
N1—H1···N4^ii^	0.88	2.16	3.0017 (14)	161

Symmetry codes: (i) −*x*, −*y*+1, −*z*+2; (ii) −*x*, −*y*+2, −*z*+1.

### (2) 3-(2H-Benzotriazol-2-yl)-2-methylpropanamide Crystal data

**Table:**

C_10_H_12_N_4_O	*Z* = 2
*M**_r_* = 204.24	*F*(000) = 216
Triclinic, *P*1	*D*_x_ = 1.329 Mg m^−^^3^
*a* = 5.5961 (11) Å	Mo *K*α radiation, λ = 0.71073 Å
*b* = 9.3462 (19) Å	Cell parameters from 5587 reflections
*c* = 10.472 (2) Å	θ = 2.1–29.2°
α = 109.83 (3)°	µ = 0.09 mm^−^^1^
β = 90.93 (3)°	*T* = 133 K
γ = 97.14 (3)°	Prism, colorless
*V* = 510.2 (2) Å^3^	0.48 × 0.33 × 0.25 mm

### (2) 3-(2H-Benzotriazol-2-yl)-2-methylpropanamide Data collection

**Table:**

Stoe IPDS 2T diffractometer	1653 reflections with *I* > 2σ(*I*)
Radiation source: fine-focus sealed tube	*R*_int_ = 0.056
Detector resolution: 6.67 pixels mm^-1^	θ_max_ = 25.0°, θ_min_ = 2.1°
area detector scans	*h* = −6→6
3731 measured reflections	*k* = −11→11
1774 independent reflections	*l* = −12→12

### (2) 3-(2H-Benzotriazol-2-yl)-2-methylpropanamide Refinement

**Table:**

Refinement on *F*^2^	Secondary atom site location: difference Fourier map
Least-squares matrix: full	Hydrogen site location: inferred from neighbouring sites
*R*[*F*^2^ > 2σ(*F*^2^)] = 0.037	H-atom parameters constrained
*wR*(*F*^2^) = 0.087	*w* = 1/[σ^2^(*F*_o_^2^) + (0.028*P*)^2^ + 0.190*P*] where *P* = (*F*_o_^2^ + 2*F*_c_^2^)/3
*S* = 1.09	(Δ/σ)_max_ < 0.001
1774 reflections	Δρ_max_ = 0.25 e Å^−^^3^
138 parameters	Δρ_min_ = −0.18 e Å^−^^3^
0 restraints	Extinction correction: SHELXL2016 (Sheldrick, 2015), Fc^*^=kFc[1+0.001xFc^2^λ^3^/sin(2θ)]^-1/4^
Primary atom site location: structure-invariant direct methods	Extinction coefficient: 0.11 (2)

### (2) 3-(2H-Benzotriazol-2-yl)-2-methylpropanamide Special details

**Table:**

Geometry. All esds (except the esd in the dihedral angle between two l.s. planes) are estimated using the full covariance matrix. The cell esds are taken into account individually in the estimation of esds in distances, angles and torsion angles; correlations between esds in cell parameters are only used when they are defined by crystal symmetry. An approximate (isotropic) treatment of cell esds is used for estimating esds involving l.s. planes.

### (2) 3-(2H-Benzotriazol-2-yl)-2-methylpropanamide Fractional atomic coordinates and isotropic or equivalent isotropic displacement parameters (Å^2^)

**Table:**

	*x*	*y*	*z*	*U*_iso_*/*U*_eq_	
C1	0.0227 (2)	0.64543 (13)	0.69653 (13)	0.0214 (3)	
C2	0.0204 (2)	0.74112 (14)	0.84610 (13)	0.0225 (3)	
H3	−0.098923	0.815190	0.857903	0.027*	
C3	0.2682 (3)	0.82849 (15)	0.90085 (13)	0.0252 (3)	
H5	0.262252	0.888303	0.998666	0.030*	
H4	0.383837	0.754250	0.892496	0.030*	
C4	−0.0516 (3)	0.63442 (17)	0.92572 (15)	0.0341 (4)	
H6	−0.215805	0.581749	0.895255	0.041*	
H8	−0.045568	0.694636	1.022969	0.041*	
H7	0.060441	0.558341	0.910078	0.041*	
C5	0.5730 (2)	1.03192 (15)	0.71230 (13)	0.0223 (3)	
C6	0.3863 (2)	1.11949 (15)	0.75999 (13)	0.0232 (3)	
C7	0.3662 (3)	1.25214 (16)	0.72735 (15)	0.0298 (3)	
H9	0.239683	1.311962	0.758654	0.036*	
C8	0.5363 (3)	1.29041 (17)	0.64894 (15)	0.0325 (4)	
H10	0.528050	1.379345	0.625352	0.039*	
C9	0.7253 (3)	1.20216 (18)	0.60133 (15)	0.0347 (4)	
H11	0.840016	1.233870	0.547020	0.042*	
C10	0.7479 (3)	1.07309 (17)	0.63095 (15)	0.0311 (4)	
H12	0.874723	1.014003	0.598479	0.037*	
N1	−0.1609 (2)	0.64790 (12)	0.61694 (11)	0.0250 (3)	
H1	−0.170803	0.591949	0.529944	0.030*	
H2	−0.273477	0.705494	0.650863	0.030*	
N2	0.5490 (2)	0.91227 (12)	0.75780 (11)	0.0239 (3)	
N3	0.3548 (2)	0.93272 (12)	0.82946 (11)	0.0220 (3)	
N4	0.2480 (2)	1.05296 (13)	0.83583 (12)	0.0258 (3)	
O	0.18732 (19)	0.56749 (11)	0.65622 (10)	0.0320 (3)	

### (2) 3-(2H-Benzotriazol-2-yl)-2-methylpropanamide Atomic displacement parameters (Å^2^)

**Table:**

	*U*^11^	*U*^22^	*U*^33^	*U*^12^	*U*^13^	*U*^23^
C1	0.0270 (7)	0.0161 (6)	0.0212 (7)	0.0033 (5)	0.0079 (5)	0.0062 (5)
C2	0.0281 (7)	0.0205 (6)	0.0194 (7)	0.0058 (5)	0.0064 (5)	0.0065 (5)
C3	0.0304 (7)	0.0262 (7)	0.0195 (6)	0.0050 (6)	0.0012 (5)	0.0084 (5)
C4	0.0497 (10)	0.0282 (7)	0.0268 (7)	0.0063 (7)	0.0146 (7)	0.0116 (6)
C5	0.0203 (7)	0.0247 (6)	0.0197 (6)	0.0020 (5)	−0.0010 (5)	0.0053 (5)
C6	0.0207 (7)	0.0240 (6)	0.0221 (7)	0.0005 (5)	0.0007 (5)	0.0050 (5)
C7	0.0279 (8)	0.0265 (7)	0.0351 (8)	0.0044 (6)	0.0017 (6)	0.0104 (6)
C8	0.0330 (8)	0.0294 (7)	0.0361 (8)	−0.0040 (6)	−0.0060 (6)	0.0157 (6)
C9	0.0273 (8)	0.0466 (9)	0.0323 (8)	−0.0058 (7)	0.0017 (6)	0.0198 (7)
C10	0.0230 (7)	0.0412 (8)	0.0296 (7)	0.0047 (6)	0.0062 (6)	0.0124 (6)
N1	0.0286 (6)	0.0252 (6)	0.0203 (6)	0.0096 (5)	0.0050 (5)	0.0042 (4)
N2	0.0226 (6)	0.0267 (6)	0.0210 (6)	0.0050 (5)	0.0029 (4)	0.0057 (4)
N3	0.0230 (6)	0.0212 (5)	0.0203 (6)	0.0029 (4)	0.0010 (4)	0.0051 (4)
N4	0.0250 (6)	0.0246 (6)	0.0276 (6)	0.0045 (5)	0.0050 (5)	0.0080 (5)
O	0.0331 (6)	0.0341 (6)	0.0240 (5)	0.0136 (5)	0.0027 (4)	0.0008 (4)

### (2) 3-(2H-Benzotriazol-2-yl)-2-methylpropanamide Geometric parameters (Å, º)

**Table:**

C1—O	1.2364 (16)	C5—C10	1.410 (2)
C1—N1	1.3199 (18)	C6—N4	1.3607 (18)
C1—C2	1.5187 (18)	C6—C7	1.4098 (19)
C2—C3	1.514 (2)	C7—C8	1.360 (2)
C2—C4	1.5255 (18)	C7—H9	0.9500
C2—H3	1.0000	C8—C9	1.415 (2)
C3—N3	1.4598 (17)	C8—H10	0.9500
C3—H5	0.9900	C9—C10	1.363 (2)
C3—H4	0.9900	C9—H11	0.9500
C4—H6	0.9800	C10—H12	0.9500
C4—H8	0.9800	N1—H1	0.8800
C4—H7	0.9800	N1—H2	0.8800
C5—N2	1.3498 (18)	N2—N3	1.3276 (16)
C5—C6	1.4013 (19)	N3—N4	1.3198 (16)
			
O—C1—N1	123.67 (12)	N4—C6—C5	108.31 (12)
O—C1—C2	119.91 (12)	N4—C6—C7	130.81 (13)
N1—C1—C2	116.40 (11)	C5—C6—C7	120.88 (13)
C3—C2—C1	110.76 (11)	C8—C7—C6	116.86 (13)
C3—C2—C4	108.52 (12)	C8—C7—H9	121.6
C1—C2—C4	108.88 (11)	C6—C7—H9	121.6
C3—C2—H3	109.6	C7—C8—C9	122.17 (13)
C1—C2—H3	109.6	C7—C8—H10	118.9
C4—C2—H3	109.6	C9—C8—H10	118.9
N3—C3—C2	112.65 (11)	C10—C9—C8	122.07 (14)
N3—C3—H5	109.1	C10—C9—H11	119.0
C2—C3—H5	109.1	C8—C9—H11	119.0
N3—C3—H4	109.1	C9—C10—C5	116.47 (14)
C2—C3—H4	109.1	C9—C10—H12	121.8
H5—C3—H4	107.8	C5—C10—H12	121.8
C2—C4—H6	109.5	C1—N1—H1	120.0
C2—C4—H8	109.5	C1—N1—H2	120.0
H6—C4—H8	109.5	H1—N1—H2	120.0
C2—C4—H7	109.5	N3—N2—C5	103.09 (11)
H6—C4—H7	109.5	N4—N3—N2	117.11 (11)
H8—C4—H7	109.5	N4—N3—C3	121.80 (11)
N2—C5—C6	108.57 (12)	N2—N3—C3	121.07 (11)
N2—C5—C10	129.88 (13)	N3—N4—C6	102.93 (11)
C6—C5—C10	121.56 (13)		
			
O—C1—C2—C3	45.53 (16)	C8—C9—C10—C5	−0.2 (2)
N1—C1—C2—C3	−136.47 (12)	N2—C5—C10—C9	−179.87 (13)
O—C1—C2—C4	−73.73 (16)	C6—C5—C10—C9	0.0 (2)
N1—C1—C2—C4	104.27 (14)	C6—C5—N2—N3	−0.24 (13)
C1—C2—C3—N3	59.74 (14)	C10—C5—N2—N3	179.62 (14)
C4—C2—C3—N3	179.22 (10)	C5—N2—N3—N4	0.10 (14)
N2—C5—C6—N4	0.30 (14)	C5—N2—N3—C3	−178.27 (11)
C10—C5—C6—N4	−179.57 (12)	C2—C3—N3—N4	65.73 (15)
N2—C5—C6—C7	−179.77 (12)	C2—C3—N3—N2	−115.97 (13)
C10—C5—C6—C7	0.4 (2)	N2—N3—N4—C6	0.08 (15)
N4—C6—C7—C8	179.50 (14)	C3—N3—N4—C6	178.44 (11)
C5—C6—C7—C8	−0.41 (19)	C5—C6—N4—N3	−0.23 (14)
C6—C7—C8—C9	0.2 (2)	C7—C6—N4—N3	179.86 (13)
C7—C8—C9—C10	0.2 (2)		

### (2) 3-(2H-Benzotriazol-2-yl)-2-methylpropanamide Hydrogen-bond geometry (Å, º)

**Table:**

*D*—H···*A*	*D*—H	H···*A*	*D*···*A*	*D*—H···*A*
N1—H1···O^i^	0.88	2.00	2.8745 (18)	170
N1—H2···N2^ii^	0.88	2.24	3.0850 (18)	161

Symmetry codes: (i) −*x*, −*y*+1, −*z*+1; (ii) *x*−1, *y*, *z*.

### (3) 3-(1H-Benzotriazol-1-yl)-N,N-dimethylpropanamide Crystal data

**Table:**

C_11_H_14_N_4_O	*Z* = 2
*M**_r_* = 218.26	*F*(000) = 232
Triclinic, *P*1	*D*_x_ = 1.342 Mg m^−^^3^
*a* = 7.1732 (6) Å	Mo *K*α radiation, λ = 0.71073 Å
*b* = 7.9945 (6) Å	Cell parameters from 6240 reflections
*c* = 9.5912 (7) Å	θ = 2.1–29.2°
α = 83.910 (6)°	µ = 0.09 mm^−^^1^
β = 86.247 (6)°	*T* = 153 K
γ = 81.528 (6)°	Block, colorless
*V* = 540.25 (7) Å^3^	0.34 × 0.32 × 0.28 mm

### (3) 3-(1H-Benzotriazol-1-yl)-N,N-dimethylpropanamide Data collection

**Table:**

Stoe IPDS 2T diffractometer	1596 reflections with *I* > 2σ(*I*)
Radiation source: fine-focus sealed tube	*R*_int_ = 0.062
Detector resolution: 6.67 pixels mm^-1^	θ_max_ = 25.0°, θ_min_ = 2.1°
area detector scans	*h* = −8→8
4194 measured reflections	*k* = −9→9
1904 independent reflections	*l* = −11→11

### (3) 3-(1H-Benzotriazol-1-yl)-N,N-dimethylpropanamide Refinement

**Table:**

Refinement on *F*^2^	Secondary atom site location: difference Fourier map
Least-squares matrix: full	Hydrogen site location: inferred from neighbouring sites
*R*[*F*^2^ > 2σ(*F*^2^)] = 0.047	H-atom parameters constrained
*wR*(*F*^2^) = 0.131	*w* = 1/[σ^2^(*F*_o_^2^) + (0.0794*P*)^2^ + 0.073*P*] where *P* = (*F*_o_^2^ + 2*F*_c_^2^)/3
*S* = 1.03	(Δ/σ)_max_ = 0.001
1904 reflections	Δρ_max_ = 0.22 e Å^−^^3^
149 parameters	Δρ_min_ = −0.20 e Å^−^^3^
0 restraints	Extinction correction: SHELXL2016 (Sheldrick, 2015), Fc^*^=kFc[1+0.001xFc^2^λ^3^/sin(2θ)]^-1/4^
Primary atom site location: structure-invariant direct methods	Extinction coefficient: 0.062 (15)

### (3) 3-(1H-Benzotriazol-1-yl)-N,N-dimethylpropanamide Special details

**Table:**

Geometry. All esds (except the esd in the dihedral angle between two l.s. planes) are estimated using the full covariance matrix. The cell esds are taken into account individually in the estimation of esds in distances, angles and torsion angles; correlations between esds in cell parameters are only used when they are defined by crystal symmetry. An approximate (isotropic) treatment of cell esds is used for estimating esds involving l.s. planes.

### (3) 3-(1H-Benzotriazol-1-yl)-N,N-dimethylpropanamide Fractional atomic coordinates and isotropic or equivalent isotropic displacement parameters (Å^2^)

**Table:**

	*x*	*y*	*z*	*U*_iso_*/*U*_eq_	Occ. (<1)
C1	0.2374 (2)	0.1609 (2)	0.89843 (17)	0.0317 (4)	
C2	0.2225 (3)	0.3473 (2)	0.91977 (18)	0.0357 (4)	
H2	0.312766	0.361341	0.990012	0.043*	
H1	0.093801	0.387131	0.957760	0.043*	
C3	0.2629 (3)	0.4566 (2)	0.78541 (18)	0.0363 (4)	
H4	0.187972	0.427798	0.710496	0.044*	
H3	0.221528	0.577397	0.800163	0.044*	
C4	0.5548 (2)	0.34481 (19)	0.63744 (16)	0.0308 (4)	
C5	0.7407 (3)	0.3710 (2)	0.64288 (17)	0.0356 (4)	
C6	0.8817 (3)	0.2937 (2)	0.5534 (2)	0.0441 (5)	
H5	1.009954	0.309393	0.557083	0.053*	
C7	0.8268 (3)	0.1947 (2)	0.46046 (19)	0.0441 (5)	
H6	0.919384	0.139249	0.398995	0.053*	
C8	0.6372 (3)	0.1724 (2)	0.45317 (18)	0.0397 (5)	
H7	0.604940	0.104362	0.385487	0.048*	
C9	0.4981 (3)	0.2455 (2)	0.54036 (17)	0.0352 (4)	
H8	0.370003	0.230034	0.535556	0.042*	
C10	0.2411 (3)	0.0869 (3)	1.15684 (19)	0.0505 (5)	
H10	0.138000	0.036749	1.209814	0.061*	
H9	0.219291	0.210450	1.159458	0.061*	
H11	0.360994	0.040258	1.198882	0.061*	
C11	0.2605 (3)	−0.1321 (2)	0.9926 (2)	0.0454 (5)	
H13A	0.386119	−0.190604	1.015826	0.054*	0.59 (2)
H14A	0.238741	−0.144173	0.894596	0.054*	0.59 (2)
H12A	0.164666	−0.182399	1.054197	0.054*	0.59 (2)
H12B	0.140231	−0.154180	0.960587	0.054*	0.41 (2)
H13B	0.287609	−0.200611	1.081816	0.054*	0.41 (2)
H14B	0.361685	−0.162385	0.922216	0.054*	0.41 (2)
N1	0.2484 (2)	0.04712 (18)	1.01231 (15)	0.0362 (4)	
N2	0.4605 (2)	0.43518 (16)	0.73920 (14)	0.0327 (4)	
N3	0.5824 (2)	0.51402 (18)	0.80163 (15)	0.0390 (4)	
N4	0.7510 (2)	0.4768 (2)	0.74587 (16)	0.0422 (4)	
O	0.2366 (2)	0.11687 (15)	0.77971 (13)	0.0442 (4)	

### (3) 3-(1H-Benzotriazol-1-yl)-N,N-dimethylpropanamide Atomic displacement parameters (Å^2^)

**Table:**

	*U*^11^	*U*^22^	*U*^33^	*U*^12^	*U*^13^	*U*^23^
C1	0.0305 (8)	0.0308 (8)	0.0359 (9)	−0.0073 (6)	−0.0002 (7)	−0.0095 (7)
C2	0.0391 (10)	0.0295 (8)	0.0404 (9)	−0.0059 (7)	0.0002 (7)	−0.0114 (7)
C3	0.0403 (10)	0.0251 (8)	0.0446 (9)	−0.0027 (7)	−0.0055 (8)	−0.0088 (7)
C4	0.0403 (9)	0.0226 (7)	0.0307 (8)	−0.0074 (7)	−0.0052 (7)	−0.0021 (6)
C5	0.0421 (10)	0.0325 (8)	0.0345 (8)	−0.0118 (7)	−0.0058 (7)	−0.0020 (7)
C6	0.0380 (10)	0.0498 (11)	0.0452 (10)	−0.0101 (8)	−0.0022 (8)	−0.0020 (8)
C7	0.0528 (12)	0.0388 (10)	0.0387 (10)	−0.0024 (8)	0.0040 (8)	−0.0046 (7)
C8	0.0592 (12)	0.0289 (8)	0.0330 (9)	−0.0102 (8)	−0.0031 (8)	−0.0066 (7)
C9	0.0453 (10)	0.0281 (8)	0.0352 (9)	−0.0111 (7)	−0.0066 (7)	−0.0064 (7)
C10	0.0641 (13)	0.0521 (12)	0.0382 (10)	−0.0184 (10)	−0.0010 (9)	−0.0050 (8)
C11	0.0473 (11)	0.0292 (9)	0.0601 (12)	−0.0079 (8)	−0.0025 (9)	−0.0032 (8)
N1	0.0405 (8)	0.0313 (7)	0.0386 (8)	−0.0088 (6)	−0.0016 (6)	−0.0065 (6)
N2	0.0418 (8)	0.0245 (6)	0.0349 (7)	−0.0102 (6)	−0.0056 (6)	−0.0071 (5)
N3	0.0482 (9)	0.0340 (7)	0.0401 (8)	−0.0164 (7)	−0.0093 (7)	−0.0090 (6)
N4	0.0446 (9)	0.0438 (9)	0.0429 (8)	−0.0166 (7)	−0.0073 (7)	−0.0092 (7)
O	0.0648 (9)	0.0330 (7)	0.0382 (7)	−0.0126 (6)	−0.0033 (6)	−0.0111 (5)

### (3) 3-(1H-Benzotriazol-1-yl)-N,N-dimethylpropanamide Geometric parameters (Å, º)

**Table:**

C1—O	1.228 (2)	C7—H6	0.9500
C1—N1	1.344 (2)	C8—C9	1.364 (3)
C1—C2	1.513 (2)	C8—H7	0.9500
C2—C3	1.515 (2)	C9—H8	0.9500
C2—H2	0.9900	C10—N1	1.451 (2)
C2—H1	0.9900	C10—H10	0.9800
C3—N2	1.448 (2)	C10—H9	0.9800
C3—H4	0.9900	C10—H11	0.9800
C3—H3	0.9900	C11—N1	1.455 (2)
C4—N2	1.361 (2)	C11—H13A	0.9800
C4—C5	1.385 (3)	C11—H14A	0.9800
C4—C9	1.401 (2)	C11—H12A	0.9800
C5—N4	1.379 (2)	C11—H12B	0.9800
C5—C6	1.399 (3)	C11—H13B	0.9800
C6—C7	1.365 (3)	C11—H14B	0.9800
C6—H5	0.9500	N2—N3	1.3535 (19)
C7—C8	1.404 (3)	N3—N4	1.296 (2)
			
O—C1—N1	121.60 (14)	N1—C10—H9	109.5
O—C1—C2	120.14 (15)	H10—C10—H9	109.5
N1—C1—C2	118.25 (14)	N1—C10—H11	109.5
C1—C2—C3	112.71 (13)	H10—C10—H11	109.5
C1—C2—H2	109.1	H9—C10—H11	109.5
C3—C2—H2	109.1	N1—C11—H13A	109.5
C1—C2—H1	109.1	N1—C11—H14A	109.5
C3—C2—H1	109.1	H13A—C11—H14A	109.5
H2—C2—H1	107.8	N1—C11—H12A	109.5
N2—C3—C2	113.14 (14)	H13A—C11—H12A	109.5
N2—C3—H4	109.0	H14A—C11—H12A	109.5
C2—C3—H4	109.0	N1—C11—H12B	109.5
N2—C3—H3	109.0	H13A—C11—H12B	141.1
C2—C3—H3	109.0	H14A—C11—H12B	56.3
H4—C3—H3	107.8	H12A—C11—H12B	56.3
N2—C4—C5	104.46 (14)	N1—C11—H13B	109.5
N2—C4—C9	133.38 (16)	H13A—C11—H13B	56.3
C5—C4—C9	122.15 (16)	H14A—C11—H13B	141.1
N4—C5—C4	108.56 (16)	H12A—C11—H13B	56.3
N4—C5—C6	130.69 (17)	H12B—C11—H13B	109.5
C4—C5—C6	120.75 (16)	N1—C11—H14B	109.5
C7—C6—C5	117.01 (17)	H13A—C11—H14B	56.3
C7—C6—H5	121.5	H14A—C11—H14B	56.3
C5—C6—H5	121.5	H12A—C11—H14B	141.1
C6—C7—C8	121.86 (18)	H12B—C11—H14B	109.5
C6—C7—H6	119.1	H13B—C11—H14B	109.5
C8—C7—H6	119.1	C1—N1—C10	125.72 (14)
C9—C8—C7	121.95 (16)	C1—N1—C11	118.52 (14)
C9—C8—H7	119.0	C10—N1—C11	115.70 (15)
C7—C8—H7	119.0	N3—N2—C4	109.63 (14)
C8—C9—C4	116.25 (16)	N3—N2—C3	119.52 (13)
C8—C9—H8	121.9	C4—N2—C3	130.85 (14)
C4—C9—H8	121.9	N4—N3—N2	109.52 (13)
N1—C10—H10	109.5	N3—N4—C5	107.81 (14)
			
O—C1—C2—C3	−16.2 (2)	C2—C1—N1—C10	2.2 (3)
N1—C1—C2—C3	164.77 (15)	O—C1—N1—C11	0.2 (3)
C1—C2—C3—N2	−72.24 (18)	C2—C1—N1—C11	179.27 (15)
N2—C4—C5—N4	−0.66 (18)	C5—C4—N2—N3	0.86 (18)
C9—C4—C5—N4	178.42 (14)	C9—C4—N2—N3	−178.07 (17)
N2—C4—C5—C6	178.83 (15)	C5—C4—N2—C3	−179.29 (15)
C9—C4—C5—C6	−2.1 (3)	C9—C4—N2—C3	1.8 (3)
N4—C5—C6—C7	−179.73 (18)	C2—C3—N2—N3	−78.81 (17)
C4—C5—C6—C7	0.9 (3)	C2—C3—N2—C4	101.35 (19)
C5—C6—C7—C8	0.8 (3)	C4—N2—N3—N4	−0.77 (19)
C6—C7—C8—C9	−1.4 (3)	C3—N2—N3—N4	179.36 (14)
C7—C8—C9—C4	0.2 (2)	N2—N3—N4—C5	0.32 (18)
N2—C4—C9—C8	−179.75 (17)	C4—C5—N4—N3	0.23 (19)
C5—C4—C9—C8	1.5 (2)	C6—C5—N4—N3	−179.20 (18)
O—C1—N1—C10	−176.82 (17)		
